# Identification of L-Cysteinamide as a Potent Inhibitor of Tyrosinase-Mediated Dopachrome Formation and Eumelanin Synthesis

**DOI:** 10.3390/antiox10081202

**Published:** 2021-07-27

**Authors:** Hyun Kyung Lee, Jae Won Ha, Yun Jeong Hwang, Yong Chool Boo

**Affiliations:** 1 Department of Molecular Medicine, Brain Korea (BK) 21 Plus Kyungpook National University (KNU) Biomedical Convergence Program, School of Medicine, Kyungpook National University, Daegu 41944, Korea; kei01116@naver.com (H.K.L.); jaewon1226@knu.ac.kr (J.W.H.); hwangkyy26@naver.com (Y.J.H.); 2 Cell and Matrix Research Institute, Kyungpook National University, Daegu 41944, Korea

**Keywords:** L-cysteinamide, tyrosinase, melanin, eumelanin, viability, MNT-1 melanoma, normal human melanocytes

## Abstract

The purpose of this study is to identify amino acid derivatives with potent anti-eumelanogenic activity. First, we compared the effects of twenty different amidated amino acids on tyrosinase (TYR)-mediated dopachrome formation in vitro and melanin content in dark-pigmented human melanoma MNT-1 cells. The results showed that only L-cysteinamide inhibited TYR-mediated dopachrome formation in vitro and reduced the melanin content of cells. Next, the antimelanogenic effect of L-cysteinamide was compared to those of other thiol compounds (L-cysteine, N-acetyl L-cysteine, glutathione, L-cysteine ethyl ester, N-acetyl L-cysteinamide, and cysteamine) and positive controls with known antimelanogenic effects (kojic acid and β-arbutin). The results showed the unique properties of L-cysteinamide, which effectively reduces melanin content without causing cytotoxicity. L-Cysteinamide did not affect the mRNA and protein levels of TYR, tyrosinase-related protein 1, and dopachrome tautomerase in MNT-1 cells. L-Cysteinamide exhibited similar properties in normal human epidermal melanocytes (HEMs). Experiments using mushroom TYR suggest that L-cysteinamide at certain concentrations can inhibit eumelanin synthesis through a dual mechanism by inhibiting TYR-catalyzed dopaquinone synthesis and by diverting the synthesized dopaquinone to the formation of DOPA-cysteinamide conjugates rather than dopachrome. Finally, L-cysteinamide was shown to increase pheomelanin content while decreasing eumelanin and total melanin contents in MNT-1 cells. This study suggests that L-cysteinamide has an optimal structure that can effectively and safely inhibit eumelanin synthesis in MNT-1 cells and HEMs, and will be useful in controlling skin hyperpigmentation.

## 1. Introduction

Melanin pigment is important not only for skin color development but also for maintaining homeostasis in various organs including the skin [1,2]. Disrupted melanin metabolism results in hypopigmentation and hyperpigmentation [3,4]. Hypopigmentation can occur when melanin synthesis is genetically or epigenetically impaired [5,6]. Hypopigmentation is caused by mutations in various genes that regulate melanocytic development (piebaldism, Waardenburg syndrome, and Tietz syndrome), melanin synthesis (oculocutaneous albinism), and melanosome transfer to keratinocytes (Hermansky-Pudlak syndrome, Chediak-Higashi syndrome, Griscelli syndrome) [7]. Hyperpigmentation can occur as a result of inflammatory reactions or aging of the skin [8,9]. Skin pigmentary disorders are important from dermatological and aesthetic points of view, and more effective and safe treatment is needed [10,11,12,13].

Several enzymes—tyrosinase (TYR), tyrosinase-related protein 1 (TYRP1), and dopachrome tautomerase (DCT)—mediate melanin synthesis in melanosomes, lysosome-related organelles in epidermal melanocytes [14,15]. TYR catalyzes the initial oxidation of L-tyrosine or L-dihydroxyphenylalanin (DOPA) to dopaquinone [16]. The subsequent reactions vary depending on the cellular contexts and result in the production of eumelanin or pheomelanin. The conjugation of dopaquinone with thiol compounds, such as L-cysteine and glutathione, and the subsequent reactions lead to the final product, reddish-yellow pheomelanin. Alternatively, dopaquinone is oxidized to dopachrome, which is then converted to 5,6-dihydroxyindole (DHI) or 5,6-dihydroxyindole-2-carboxylic acid (DHICA). Polymerization of DHI, DHICA, and their quinones lead to the production of brownish-black eumelanin [17].

There are multiple targets for the artificial regulation of skin pigmentation [18]. These include the receptors on the membrane of melanocytes, proteins that transmit intracellular signals, and melanogenic enzymes within melanocytes, and proteins involved in melanosome biogenesis. Many natural and synthetic are known to directly inhibit the catalytic activity of TYR [19,20,21,22].

Kahn et al. [23] tested various L-amino acids against the ortho-dihydroxyphenolase activity of mushroom TYR. L-Lysine, L-glycine, L-histidine, and L-phenylalanine inhibited enzyme activity by 50% at 50, 65, 120, and 200 mM, respectively, whereas L-alanine, L-proline, L-serine, L-isoleucine, L-leucine, L-asparagine, L-valine, DL-aspartic acid, L-glutamic acid, and L-tryptophan were ineffective. The highest inhibitory effect was exhibited by L-cysteine which completely suppressed dopachrome formation at 0.3 mM. Tseng et al. [24] tested 20 × 20 dipeptides against mushroom TYR activity. Cysteine-containing dipeptides, especially N-terminal cysteine-containing dipeptides, exhibited more potent inhibitory effects compared to other dipeptides. Luisi et al. [25] tested various sulfur-containing amino acids and tripeptides against mushroom TYR-mediated dopachrome formation. L-Cysteine, L-cystine, γ-oxa-glutamyl analog of glutathione, and ergothioneine exhibited stronger inhibitory effects compared to glutathione, whereas taurine exhibited a slightly weaker inhibitory effect. Overall, TYR-mediated dopachrome formation in eumelanin synthesis was inhibited by various thiol compounds, and this effect is attributed to the formation of a conjugate between dopaquinone and each thiol compound [26,27].

In anticipation of the potential depigmenting effects of certain amino acids, peptides, and their derivatives, we aimed to discover small molecular amino acid derivatives that have potent anti-eumelanogenic activity and high safety. In the present study, we first tested various amidated L-amino acids against eumelanin synthesis in human melanoma MNT-1 cells. As L-cysteinamide showed the most potent inhibition, its activity was further compared with those of other thiol compounds (L-cysteine, N-acetyl L-cysteine, glutathione, L-cysteine ethyl ester, N-acetyl L-cysteinamide, cysteamine). Kojic acid and β-arbutin were used as positive controls with antimelanogenic effects [28,29]. As a result, L-cysteinamide was suggested to have an optimal structure to inhibit cellular eumelanin synthesis.

## 2. Materials and Methods 

### 2.1. Materials

Twenty kinds of amidated amino acids (mostly in hydrochloride salt forms except for L-glutamic acid α-amide) were purchased from Watanabe Chemical Ind., Ltd. (Hiroshima, Japan). They are listed in Table 1. L-Tyrosine, L-DOPA, kojic acid, β-arbutin, L-cysteine, glutathione, N-acetyl L-cysteine, N-acetyl L-cysteinamide, L-cysteine ethyl ester hydrochloride, cysteamine, and pyrocatechol violet (PCV) were purchased from Sigma-Aldrich (St. Louis, MO, USA).

### 2.2. Cell Culture

The MNT-1 human melanoma cells were obtained from American Type Culture Collection (Manassas, VA, USA), and routinely cultured in Dulbecco’s modified Eagle’s medium supplemented with 20% (*v*/*v*) fetal bovine serum (Gibco BRL, Grand Island, NY, USA), 10% (*v*/*v*) AIM-V medium (ThermoFisher Scientific, Waltham, MA, USA), 1% (*v*/*v*) nonessential amino acids (ThermoFisher Scientific), and antibiotics (100 U∙mL^−1^ penicillin, 0.1 mg∙mL^−1^ streptomycin, 0.25 μg∙mL^−1^ amphotericin B) (Thermo Fisher). Cells were maintained on the T-75 flask (SPL Life Science, Gyeonggi-do, South Korea) at 37 °C in a humidified atmosphere of 5% CO_2_ and 95% air.

Human epidermal melanocytes (HEMs), derived from moderately pigmented neonatal foreskins, were purchased from Cascade Biologics (Portland, OR, USA). Cells were maintained on the T-25 flask (SPL Life Science) at 37 °C in a humidified atmosphere of 5% CO_2_ and 95% air. Cells were grown in Medium 254 supplemented with human melanocyte growth supplement (Cascade Biologics), and antibiotics (100 U∙mL^−1^ penicillin, 0.1 mg∙mL^−1^ streptomycin, 0.25 μg∙mL^−1^ amphotericin B) (Thermo Fisher).

### 2.3. Cell Viability Assay

Cell viability was measured by using 3-(4,5-dimethythiazol-2-yl) 2,5-diphenyltetrazolium bromide (MTT) (Amresco, Solon, OH, USA) [30]. MTT was dissolved in phosphate-buffed saline (PBS) (5 mg∙mL^−1^) and filtered through a 0.22 μm membrane filter (Corning Inc., Corning, NY, USA). Typically, cells were cultured in a 96-well plate (1.5 × 10^4^ cells per well) for 24 h and then treated with the test substance for 48 h. Cells were then washed with PBS and incubated in 100 μL culture medium containing 1.0 mg∙mL^−1^ MTT for 3 h at 37 °C. The medium was discarded by suction, and the formazan dye accumulated in the cells was extracted with 100 μL dimethyl sulfoxide (DMSO). The absorbance at 570 nm (A_570_) was measured using a Spectrostar Nano microplate reader (BMG LABTECH GmbH, Ortenberg, Germany).

### 2.4. Assay for Human TYR Activity

TYR activity (TYR-mediated dopachrome formation) was determined by spectrophotometry using L-tyrosine and L-DOPA as substrates [31,32]. In experiments examining the effects of various substances on TYR catalytic activity in vitro, a lysate of human embryonic kidney 293 cells constitutively expressing human TYR (HEK293-TYR) was used as a sole TYR preparation in the entire assay [33]. In experiments examining the effects of various substances on the level of TYR activity in MNT-1 cells or HEMs, the cells treated with each substance provided different cell lysates. Then, assays were performed to compare TYR activity between different cell lysates. Cell lysis buffer consisted of 10 mM Tris-HCl buffer (pH 7.4), 120 mM NaCl, 25 mM KCl, 2.0 mM EDTA, 1.0 mM EGTA, 0.5% Triton X-100, and a protease inhibitor cocktail (Roche, Mannheim, Germany) at 4 °C. Cells were lysed and centrifuged at 13,000 rpm for 15 min at 4 °C to obtain the supernatants as cell lysates. Protein content was determined using the Bio-Rad DC assay (Bio-Rad Laboratories, Hercules, CA, USA). The reaction mixture for TYR activity assay (200 μL) consisted of 100 mM sodium phosphate (pH 6.8), a cell lysate (40 μg protein), a test substance (if any), 1.0 mM L-tyrosine, and 42 μΜ L-DOPA. The reaction mixture was incubated at 37 °C, and dopachrome formation was monitored by absorbance at 475 nm (A_475_) for 8 h at 1 h intervals. TYR activity was expressed as % of the control value.

### 2.5. Melanin Content Assay

MNT-1 cells were plated in 96-well plates (1.5 × 10^4^ cells per well) and maintained in a growth medium for 24 h. The cells were treated with L-cysteinamide or other test substances at the indicated concentration for 72 h. Cells were washed with cold PBS twice and total melanin was extracted with 120 μL of 1.0 NaOH/10% DMSO solution at 60 °C for 30 min. HEMs were seeded in 6-well plates (10^5^ cells per well) and cultured in a growth medium for 24 h. The cells were treated with L-cysteinamide at the indicated concentrations for 72 h. Then cells were subjected to the total melanin content assay as above using 200 μL of 1.0 NaOH/10% DMSO solution at 60 °C for 30 min. The melanin extracts were then centrifuged at 13,000 rpm for 15 min at 4 °C. The absorbance at 400 nm (A_400_) of the supernatants was measured using a Spectrostar Nano microplate reader (BMG LABTECH GmbH, Ortenberg, Germany) and normalized to the protein content.

In experiments to estimate the pheomelanin and eumelanin contents, MNT-1 cells were cultured in T-75 flasks and treated with 1 mM L-cysteinamide for 72 h. Pheomelanin extraction was performed as in [34]. Cells from two T-75 flasks (ca. 1.6 × 10^7^ cells) were extracted with 200 μL of 0.1 M sodium phosphate buffer (pH 10.5) at 25 °C for 10 min. After centrifugation as above, the supernatant was partitioned with 200 μL chloroform to remove fatty impurities and the resulting aqueous layer was used as the pheomelanin extract. The pheomelanin-depleted pellet was extracted with 200 μL of 1.0 M NaOH/10% DMSO solution at 60 °C for 30 min, centrifuged, and the supernatant was used as the eumelanin extract. The A_400_ of pheomelanin extract or ten-fold diluted eumelanin extract was measured and corrected for protein content, dilution factor, and molar extinction coefficients [35].

### 2.6. Quantitative Reverse Transcription-Polymerase Chain Reaction (qRT-PCR) Analysis

Cellular mRNA was extracted using the RNeasy kit (Qiagen, Valias, CA, USA), and complementary DNA (cDNA) was synthesized using a high-capacity cDNA archive kit (Applied Biosystems, Foster City, CA, USA). The qRT-PCR analysis was performed with the StepOnePlus™ real-time PCR system (Applied Biosystems). The reaction mixture (20 µL) consisted of SYBR^®^ Green PCR Master Mix (Applied Biosystems), cDNA (60 ng), and gene-specific primer sets (2 pmol) (Macrogen, Seoul, Korea). The primer sequences are shown in Table 2.

Thermal cycling parameters for PCR were set as follows: initial incubation at 50 °C for 2 min, DNA polymerase activation at 95 °C for 10 min, 40 amplification cycles (annealing and extension at 60 °C for 1 min and melting at 95 °C for 15 s). Melting curves were generated to verify the homogeneity of the amplified products. The mRNA levels of TYR, TYRP1, and DCT were normalized to that of an internal standard, glyceraldehyde 3-phosphate dehydrogenase (GAPDH) using the relative Ct method. Ct is defined as the number of cycles required for the PCR signal to exceed the threshold. Fold changes in the test group compared to the control group were calculated as 2^−^^ΔΔ^^Ct^, where ΔΔCt = ΔCt_(test)_ − ΔCt_(control)_ = [Ct_(gene, test)_ − Ct_(reference, test)_] − [Ct_(gene, control)_ − Ct_(reference, control)_].

### 2.7. Western Blotting

Western blotting was performed as previously described [39]. An antibody to TYR (#127217) was purchased from MyBioSource (San Diego, CA, USA). Antibodies against TYRP1 (#10443) and β-actin (#47778) were purchased from Santa Cruz Biotechnology (Santa Cruz, CA, USA). An antibody against DCT (#74073) was purchased from Abcam (Cambridge, UK). An anti-rabbit IgG (#2357) antibody conjugated to horseradish peroxidase (HRP) was purchased from Santa Cruz Biotechnology. An anti-mouse IgG (#7076) antibody conjugated to HRP was purchased from Cell Signaling Technology (Danvers, MA, USA). Each antibody was diluted 1 to 1000 in antibody dilution buffer containing 20 mM Tris-Cl (pH 7.5), 200 mM NaCl, and 5% skim milk.

Proteins in cell lysate samples were denatured by adding Laemmli 5 × sample buffer and heating at 95 °C for 5 min. Proteins (20 μg) were resolved with 7.5% SDS-polyacrylamide gel electrophoresis at 100 V and electrically transferred to a polyvinylidene difluoride membrane (Amersham Pharmacia, Little Chalfont, UK) at 4 °C overnight. After blocking incubation with a 5% skim milk solution, the membrane was incubated with the primary antibody at 4 °C overnight, followed by incubation with the secondary antibody at room temperature for 1 h. The target protein bands were visualized with a chemiluminescence method using the picoEPD Western Reagent kit (ELPIS-Biotech, Daejeon, Korea). The captured blot images were analyzed using the NIH Image J program.

### 2.8. Fontana-Masson Staining

Cells were fixed in 4% p-formaldehyde for 10 min at room temperature, and the melanin in cells was highlighted using a Fontana-Masson staining kit from American Master*Tech Scientific, Inc. (Lodi, CA, USA). Briefly, cells were incubated with an ammoniacal silver solution for 10 min at 60 °C, followed by incubations in a 0.1% gold chloride solution and then in a 5% sodium thiosulfate solution. Cell morphology and pigmentation were examined under a phase-contrast microscope (Eclipse TS100, Nikon Instruments Inc., Melville, NY, USA).

### 2.9. Assay for Mushroom TYR Activity (TYR-Mediated Dopachrome Formation)

The assay mixture consisted of 100 mM sodium phosphate buffer (pH 6.8), an inhibitor at varied concentrations (0, 100, 200, or 300 μM), L-DOPA at varied concentrations (0.33, 0.5, 1.0 or 2.0 mM), and 25 units mL^−1^ mushroom TYR (Sigma-Aldrich). The reaction mixture was incubated at 37 °C, and A_475_ was measured for 5 min. The concentration of dopachrome produced was calculated using a molar extinction coefficient for dopachrome of ε = 3700 M^−1^ cm^−1^ [9]. The enzyme-catalyzed reaction velocities were corrected for the effects of nonenzymatic reactions. The degree of inhibition was calculated as follows: degree of inhibition (%) = (A − B)/A × 100, where A and B are the corrected reaction velocities in the absence and presence of an inhibitor, respectively. In an additional experiment, reaction mixtures containing 100 mM sodium phosphate buffer (pH 6.8), 2 mM L-DOPA, 25 units mL^−1^ mushroom TYR, and L-cysteinamide at 0, 100, 200, or 300 μM was incubated at 37 °C and UV-visible spectrum of reaction was measured for 15 min at 1 min-intervals by a Shimadzu UV-1650PC spectrophotometer (Shimadzu Corporation, Kyoto, Japan).

### 2.10. Assay for Copper Chelating Activity

The copper-chelating activity was assessed by a spectrometric method using PCV [40]. An aqueous solution of 200 μM PCV was incubated at 25 °C for 20 min in the absence and presence of 200 μM CuSO_4_ and a test substance, and the absorption spectrum was recorded using a Shimadzu UV-1650PC spectrophotometer. The copper chelating activity was evaluated by the absorbance at 632 nm (A_632_) of the [Cu^2+^-PCV] complex.

### 2.11. Statistical Analysis

The experimental results are presented as mean ± standard deviation (SD) of at least three independent experiments. Data were analyzed using SigmaStat v.3.11 Statistical Analysis Software (Systat Software Inc, San Jose, CA, USA). The presence of significantly different group means was determined at the *p* < 0.05 level using one-way analysis of variance (ANOVA). Each experimental group was compared with the control group using Dunnett’s test, and statistical significance was expressed as * *p* < 0.05 or ** *p* < 0.01. All groups were compared to each other using Duncan’s multiple range test and different alphabetic letters were used to represent statistically different means.

## 3. Results

### 3.1. Effects of Various Amidated Amino Acids on TYR Activity (TYR-Mediated Dopachrome Formation) In Vitro and Melanin Content of MNT-1 Cells

**Figure 1:**
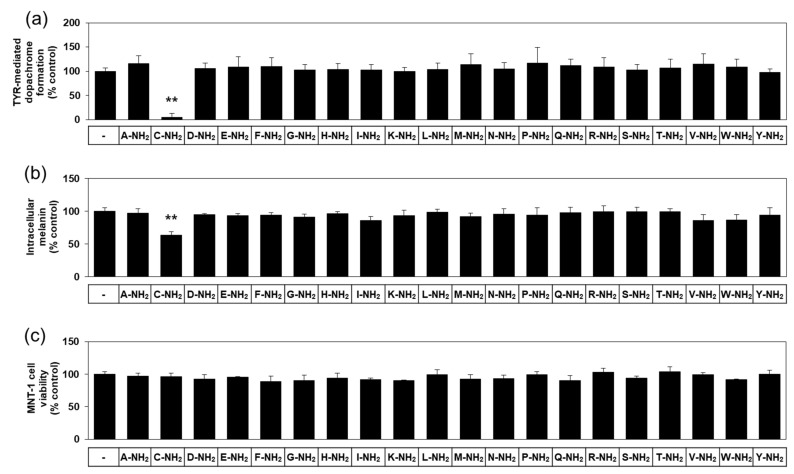
Effects of twenty different amidated amino acids on tyrosinase (TYR)-mediated dopachrome formation in vitro, and the eumelanin content and viability of MNT-1 cells. In (**a**), TYR-mediated dopachrome formation was determined using a lysate of human embryonic kidney 293 cells constitutively expressing human TYR in the absence (the control) or presence of each substance at 0.2 mM. Data are presented as % of control (means ± SD, *n* = 4). ** *p* < 0.01 vs. control. In (**b**) and (**c**), MNT-1 cells were treated with vehicle (control) or each substance at 0.5 mM for 72 h. Melanin content (**b**) and cell viability (**c**) are presented as % of control (means ± SD, *n* = 4). ** *p* < 0.01 vs. control.

There are twenty different natural L-amino acids used in protein synthesis in cells. The effects of various free L-amino acids on TYR-catalyzed reactions have been investigated previously [23]. In anticipation of different biochemical properties between free and amidated amino acids, we used various amidated L-amino acids as test substances in the present study. The first experiment compared twenty different amidated L-amino acids for their effect on human TYR-mediated dopachrome formation in vitro. As can be seen in Figure 1a, L-cysteinamide (200 μM) markedly inhibited TYR-mediated dopachrome formation, while other amidated amino acids had no significant effect.

In the subsequent experiments, darkly pigmented human melanoma MNT-1 cells were treated with each of the twenty different amidated amino acids to compare their effects on cellular melanin synthesis. As can be seen in Figure 1b, L-cysteinamide (500 μM) significantly reduced the melanin content in MNT-1 cells, but the other nineteen amidated amino acids did not have such an effect. All amidated amino acids tested did not significantly affect the viability of MNT-1 cells (Figure 1c). Therefore, L-cysteinamide was identified as a potent regulator of melanin synthesis in cells.

### 3.2. Effects of L-Cysteinamide and Other Thiol Compounds on TYR Activity (TYR-Mediated Dopachrome Formation) In Vitro and Melanin Content of MNT-1 Cells

Does L-cysteinamide have any advantageous properties in terms of activity or safety compared to other antimelanogenic compounds? We addressed this question by comparing the activities of L-cysteinamide with those of other thiol compounds, such as L-cysteine, N-acetyl L-cysteine, and glutathione, as well as positive controls (kojic acid and β-arbutin).

**Figure 2:**
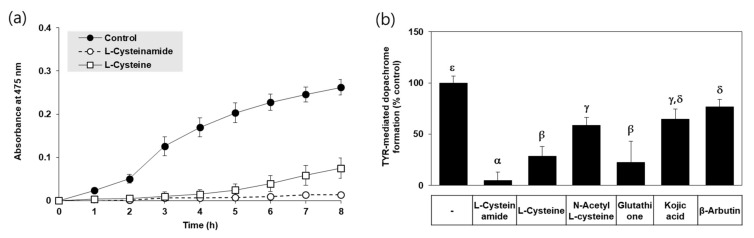
Effects of L-cysteinamide, L-cysteine, N-acetyl L-cysteine, glutathione, kojic acid, and β-arbutin on TYR-mediated dopachrome formation in vitro. (**a**) Time-dependent changes in A_475_ of the TYR reaction mixture in the absence (control) or presence of L-cysteinamide or L-cysteine at 0.2 mM (*n* = 3). (**b**) TYR-mediated dopachrome formation determined in the absence (control) and presence of each substance at 0.2 mM (*n* = 4). Data are presented as % of control (means ± SD). Duncan’s multiple range test was performed to compare all group means to each other. Groups that share the same Greek letters (α, β, γ, δ, or ε) do not have significantly different means at the *p* < 0.05 level.

In an in vitro assay, TYR-mediated dopachrome formation was monitored by A_475_ in the absence and presence of each compound. Typical kinetic profiles of the reaction are shown in Figure 2a. The A_475_ of the control reaction mixture gradually increased in a time-dependent manner indicating TYR-mediated dopachrome formation, and that change was almost completely suppressed by L-cysteinamide (200 μM). L-cysteine also inhibited the change to a lower degree than L-cysteinamide. As shown in Figure 2b, L-cysteinamide showed the most potent inhibitory activity against TYR activity (TYR-mediated dopachrome formation), followed by glutathione, L-cysteine, N-acetyl L-cysteine, and kojic acid, and β-arbutin.

**Figure 3:**
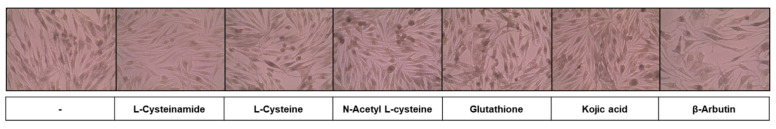
Effects of L-cysteinamide, L-cysteine, N-acetyl L-cysteine, glutathione, kojic acid, and β-arbutin on the morphology and pigmentation of MNT-1 cells. Cells were treated with each substance at 1.0 mM for 72 h and subjected to Fontana-Masson staining.

In the subsequent experiments, MNT-1 cells were treated with various thiol compounds and positive controls (kojic acid and β-arbutin) to examine their effects on cellular melanin synthesis and cell viability. To visualize melanin accumulation, we observed cells under a microscope after Fontana-Masson staining. As can be seen in Figure 3, L-cysteinamide (1.0 mM) did not significantly affect cell morphology while reducing cell pigmentation. L-Cysteine, N-acetyl L-cysteine, glutathione, and kojic acid did not significantly affect cell morphology. β-Arbutin induced enlarged cell shapes which are usually observed in senescing cells (probably due to the toxic action of hydroquinone).

**Figure 4:**
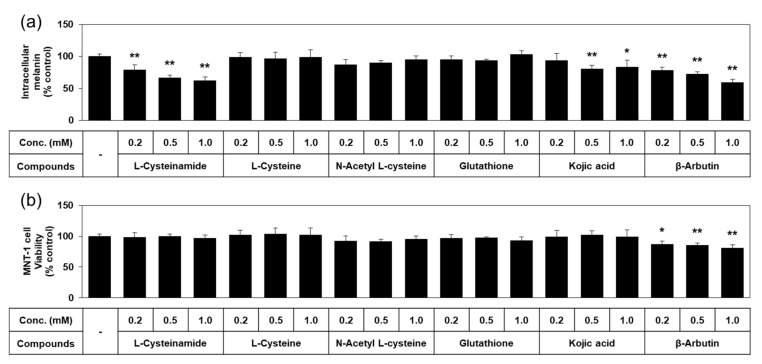
Effects of L-cysteinamide, L-cysteine, N-acetyl L-cysteine, glutathione, kojic acid, and β-arbutin on the melanin content and viability of MNT-1 cells. Cells were treated with vehicle (control) or each substance at the indicated concentrations for 72 h. Melanin content (**a**) and cell viability (**b**) are presented as % of control (means ± SD, *n* = 4). * *p* < 0.05 vs. control; ** *p* < 0.01 vs. control.

Quantified data on melanin content and viability of MNT-1 cells treated with each of the thiol compounds and known TYR inhibitors are shown in Figure 4. L-cysteinamide dose-dependently reduced the melanin content of MNT-1, without causing cell viability loss. L-Cysteine, N-acetyl L-cysteine, and glutathione did not exhibit such antimelanogenic effects or cytotoxic effects. Kojic acid slightly reduced the melanin content without cytotoxic effect. β-Arbutin decreased the melanin content as much as L-cysteinamide, but significantly reduced cell viability. Therefore, L-cysteinamide was proposed as a highly effective and safe antimelanogenic agent compared to other tested compounds.

**Figure 5:**
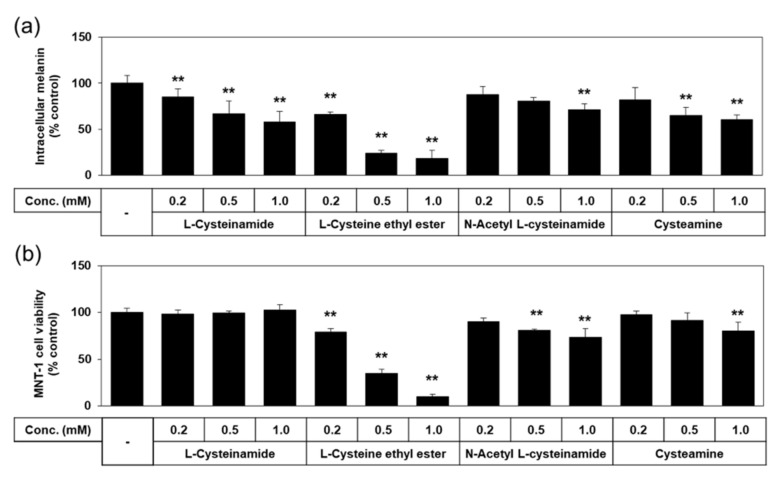
Effects of L-cysteinamide, L-cysteine ethyl ester, N-acetyl L-cysteinamide, and cysteamine on the melanin content and viability of MNT-1 cells. Cells were treated with vehicle (control) or each substance at the indicated concentrations for 72 h. Melanin content (**a**) and cell viability (**b**) are presented as % of control (means ± SD, *n* = 3). ** *p* < 0.01 vs. control.

We tested additional thiol compounds, such as L-cysteine ethyl ester, N-acetyl L-cysteinamide, and cysteamine, which are presumed to be more hydrophobic or cell-permeable than L-cysteinamide or L-cysteine. As shown in Figure 5, the antimelanogenic effect of L-cysteinamide was reproduced in this experiment by reducing the melanin content of MNT-1 cells without decreasing cell viability at 0.2–1.0 mM. L-Cysteine ethyl ester and N-acetyl L-cysteinamide lowered melanin contents at certain concentrations but these effects were ascribed to their cytotoxicities. Cysteamine lowered melanin content without reducing cell viability at 0.5 mM, but induced cytotoxicity at 1.0 mM. Therefore, none of these thiol compounds were considered to have more advantageous properties compared to L-cysteinamide.

### 3.3. Effects of L-Cysteinamide on the Expression Levels Melanogenic Enzymes in MNT-1 Cells

Can L-cysteinamide suppress cellular melanin synthesis through mechanisms other than inhibition of TYR-mediated dopachrome formation? To address this issue, we examined the effects of L-cysteinamide on the cellular TYR activity level as well as the mRNA and protein expression levels of melanogenic enzymes in MNT-1 cells, compared to other thiol compounds (L-cysteine, N-acetyl L-cysteine, and glutathione) and positive controls (kojic acid and β-arbutin).

**Figure 6:**
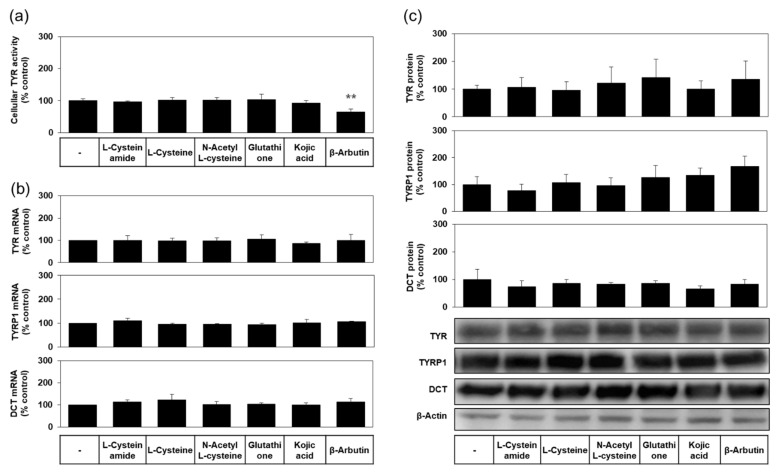
Effects of L-cysteinamide, L-cysteine, N-acetyl L-cysteine, glutathione, kojic acid, and β-arbutin on the activity levels of TYR, and the mRNA and protein expression levels of TYR, tyrosinase-related protein-1 (TYRP1), dopachrome tautomerase (DCT). MNT-1 cells were treated with vehicle (control) or each substance at 1.0 mM for 24 h. (**a**) TYR activity was determined using cell lysates (*n* = 4). (**b**) The mRNA levels of TYR, TYRP1, and DCT were determined by qRT-PCR and normalized to glyceraldehyde 3-phosphate dehydrogenase (GAPDH) mRNA levels (*n* = 3). (**c**) Their protein levels were quantified by Western blot and normalized to β-actin protein levels (*n* = 4). Typical blot images are shown. Data are presented as % of control (means ± SD). ** *p* < 0.01 vs. control.

As shown in Figure 6a, the level of cellular TYR activity was not reduced by L-cysteinamide, indicating irreversible inactivation of TYR did not occur. As can be seen in Figure 6b,c, the mRNA and protein expression levels of TYR, TYRP1, and DCT were not affected by L-cysteinamide. Therefore, L-cysteinamide is considered to suppress cellular melanin synthesis without irreversible inactivation of TYR enzyme or down-regulation of melanogenic enzyme expression.

Other compounds, such as L-cysteine, N-acetyl L-cysteine, glutathione, and kojic acid did not affect the levels of cellular TYR activity (Figure 6a), and the mRNA and protein expression levels of TYR, TYRP1, and DCT (Figure 6b,c). β-Arbutin did not affect the mRNA and protein expression levels of TYR, TYRP1, and DCT (Figure 6b,c), but significantly lowered the level of cellular TYR activity (Figure 6a), suggesting that this compound could inactivate the TYR enzyme in cells.

### 3.4. Mechanism Study Using Mushroom TYR

**Figure 7:**
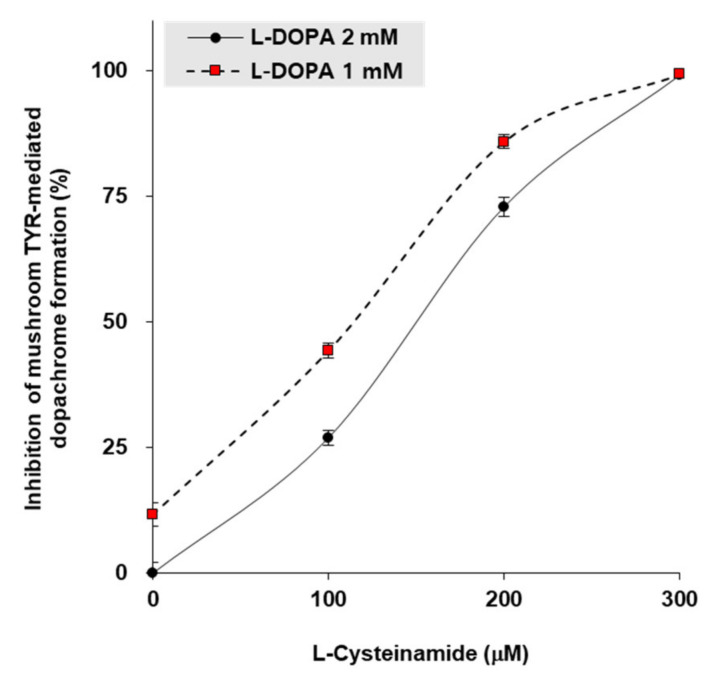
Inhibition of mushroom TYR-mediated dopachrome formation by L-cysteinamide. Mushroom TYR-mediated dopachrome formation was measured at varying concentrations of L-dihydroxyphenylalanin (DOPA) and L-cysteinamide. Data are presented as mean ± SD (*n* = 4).

To gain insight into the mechanism by which L-cysteinamide inhibits TYR, in vitro experiments were performed using mushroom TYR. When L-DOPA concentrations were 1 mM or 2 mM, the 50% inhibitory concentrations (IC_50_) of L-cysteinamide were 115 μM and 150 μM, respectively (Figure 7).

**Figure 8:**
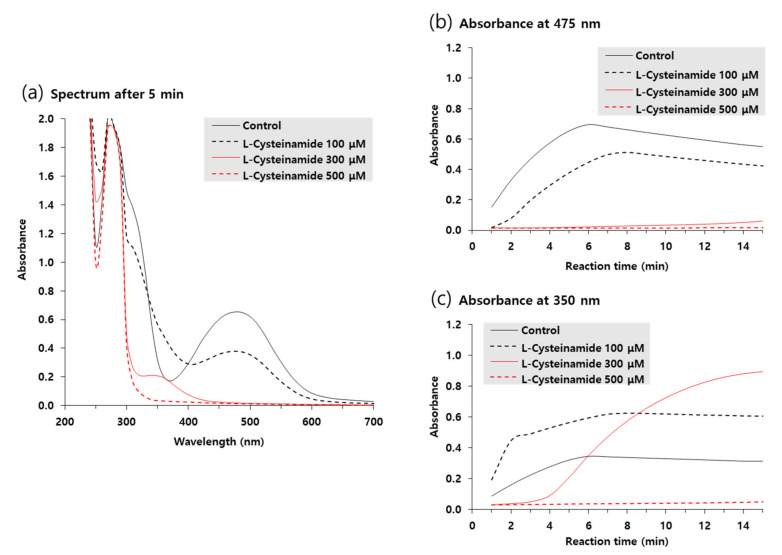
Ultraviolet-visible absorption spectrum changes during TYR-catalyzed reactions. The reaction mixture containing 2 mM L-DOPA and varied concentrations of L-cysteinamide (100, 300, or 500 μM) were incubated at 25 °C for the indicated time. Spectra after 5-min reaction are shown in (**a**). Time-dependent changes in A_475_ and A_350_ are shown in (**b**) and (**c**), respectively.

Ultraviolet-visible absorption spectra of reaction mixtures incubated for 5 min in the absence or presence of various concentrations of L-cysteinamide were compared in Figure 8a. The A_475_ due to accumulated dopachrome was highest in the control reaction mixture and was dose-dependently decreased by L-cysteinamide. In contrast, the absorbance at 350 nm (A_350_) increased by 100 μM L-cysteinamide, implying the formation of DOPA-cysteinamide conjugates.

Figure 8b,c shows the time-dependent changes in A_475_ and A_350_ of the reaction mixtures, respectively. In the absence of L-cysteinamide, both A_475_ and A_350_ increased in a similar pattern and reached a plateau after 6 min. In the presence of 100 μM L-cysteinamide, the increase in A_475_ was partially suppressed, but A_350_ rapidly increased at the initial time point and then gradually increased to a plateau, indicating that the synthesized dopaquinone was quickly captured by L-cysteinamide to form DOPA-cysteinamide conjugates. In the presence of 300 μM L-cysteinamide, the change in A_475_ was almost completely suppressed, and A_350_ at the initial time points was lower than the value observed with 100 μM L-cysteinamide, indicating a greater inhibition of TYR activity. Under this condition, A_350_ increased slowly and steadily, indicating that the dopaquinone was synthesized at a low rate and was continuously captured by L-cysteinamide to generate DOPA-cysteinamide conjugates, preventing dopachrome formation. There was no increase in both A_475_ and A_350_ in the presence of 500 μM L-cysteinamide, indicating that TYR activity was completely inhibited, and neither DOPA-cysteinamide conjugates nor dopachrome could be formed.

### 3.5. Copper Chelating Activity of L-Cysteinamide

**Figure 9:**
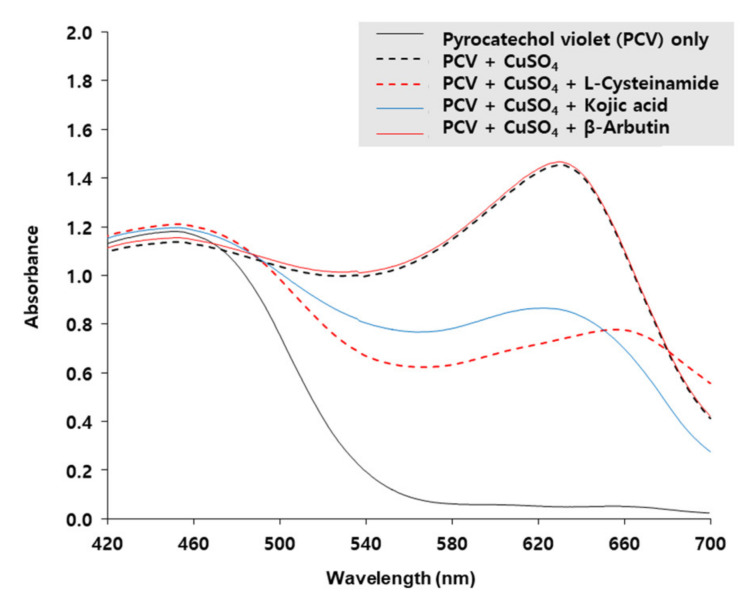
Copper-chelating activities of L-cysteinamide, kojic acid, and β-arbutin. Absorption spectra of 200 μM pyrocatechol violet (PCV) reacted with 200 μM CuSO_4_ in the absence or presence of L-cysteinamide, kojic acid, or β-arbutin, each at 200 μM, are shown.

The copper-chelating activity of L-cysteinamide was determined by using PCV which is complexed with Cu^2+^ ion to form a chromogen with the maximum absorption at 632 nm [40]. As shown in Figure 9, the A_632_ of PCV was increased by CuSO_4_, and the change was reduced by L-cysteinamide and kojic acid, but not affected by β-arbutin, indicating that both L-cysteinamide and kojic acid have potent copper chelating activities. The results of this experiment suggested that the catechol-structured compound and L-cysteinamide could compete for the Cu^2+^ ion of the active site of TYR.

### 3.6. Effects of L-Cysteinamide on the Pheomelanin and Eumelanin Contents of MNT-1 Cells

**Figure 10:**
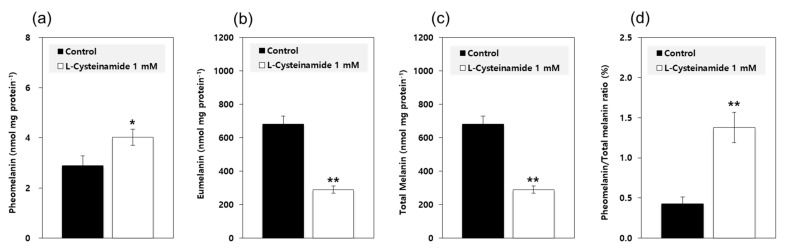
Effects of L-cysteinamide on the pheomelanin and eumelanin contents of MNT-1 cells. Cells were treated with vehicle (control) or 1 mM L-cysteinamide for 72 h. The contents of pheomelanin (**a**) and eumelanin (**b**) were separately estimated and used to calculate the total melanin contents (**c**) and the pheomelanin to total melanin ratios (**d**). Data are presented as means ± SD (*n* = 3). * *p* < 0.05 vs. control; ** *p* < 0.01 vs. control.

Based on the results from the mechanistic study using mushroom TYR, it was predicted that L-cysteinamide at certain concentrations would increase the pheomelanin synthesis and decrease the eumelanin synthesis. To examine this possibility, MNT-1 cells were treated with 1 mM L-cysteinamide and the contents of pheomelanin and eumelanin were separately estimated. As shown in Figure 10, L-cysteinamide increased the pheomelanin content while decreasing the eumelanin content and total melanin content. It also increased the pheomelanin to total melanin ratios from 0.43% in control cells to 1.38% in L-cysteinamide-treated cells.

### 3.7. Effects of L-Cysteinamide on the Melanin Content, the Cellular TYR Activity, and the Expression Levels of Melanogenic Enzymes in Normal HEMs

**Figure 11:**
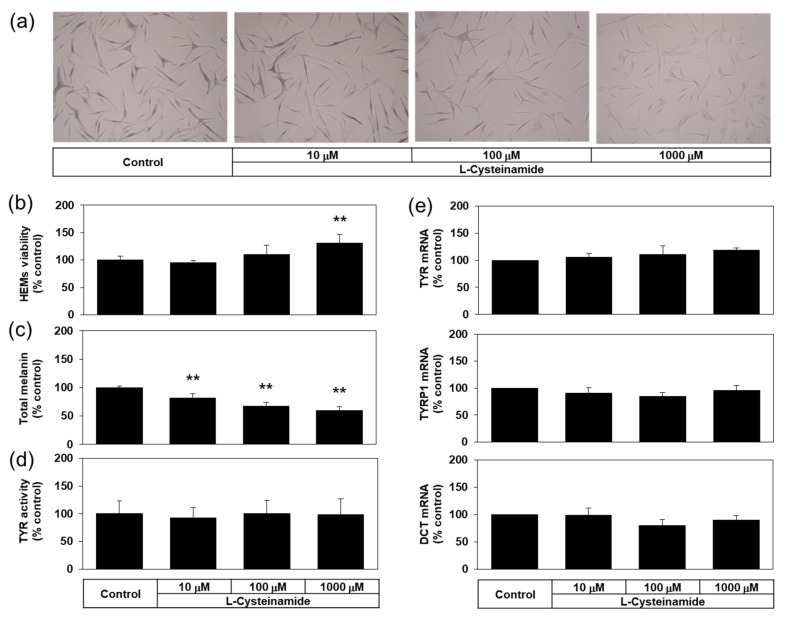
Effects of L-cysteinamide on the viability, the melanin content, the cellular TYR activity, and the mRNA expression levels of melanogenic enzymes in normal human epidermal melanocytes (HEMs). Cells treated with vehicle (control) or L-cysteinamide at the specified concentration for 72 h (**a**–**c**) or 24 h (**d**,**e**). Cells were subjected to Fontana-Masson staining (**a**), and assays to determine total melanin content (*n* = 4) (**b**) and viability (*n* = 4) (**c**). Cellular TYR activity was determined using cell lysates (*n* = 4) (d). The mRNA levels of TYR, TYRP1, and DCT were determined by qRT-PCR and normalized to GAPDH mRNA levels (*n* = 3) (**e**). Data are presented as % of control (means ± SD). ** *p* < 0.01 vs. control.

Many experiments in the present study were conducted using a darkly pigmented human melanoma MNT-1 cell line for its ability to constitutively produce a large amount of melanin. Then, does L-cysteinamide inhibit melanin synthesis only in this particular cell line? To address this question, the antimelanogenic effect of L-cysteinamide was further examined in normal HEMs. As can be seen in Figure 11a,b, L-cysteinamide showed hypopigmenting effects and reduced melanin content in normal HEMs in a dose-dependent manner. L-cysteinamide did not show cytotoxic effects and rather slightly increased cell viability at 1 mM compared to the control (Figure 11c). The level of cellular TYR activity (Figure 11d), and the mRNA expression levels of TYR, TYRP1, and DCT (Figure 11e) were not affected by L-cysteinamide up to 1 mM. Therefore, L-cysteinamide is considered to inhibit cellular melanin synthesis without irreversible inactivation of TYR enzyme or down-regulation of melanogenic enzyme expression in HEMs.

## 4. Discussion

Certain amino acids and peptides stimulate or inhibit cellular melanin synthesis and might be useful for artificially up- or down-regulating skin pigmentation for cosmetic or therapeutic purposes [18]. Here, we found that L-cysteinamide was the unique amidated L-amino acid with potent inhibitory effects on the TYR-mediated dopachrome formation in vitro and the eumelanin synthesis in MNT-1 cells. In addition, the activity of L-cysteinamide was superior to those of many other thiol compounds.

Previous studies showed that various thiol compounds, such as L-cysteine and glutathione, inhibited TYR-mediated reactions in vitro [25]. Among natural L-amino acids, L-cysteine most potently inhibited TYR-mediated browning in vitro [23]. Deprivation of L-cysteine promoted eumelanin synthesis in human melanoma cells [41]. In addition, the pheomelanin/total melanin ratio in melanocytes cultured in a high L-tyrosine medium increased with L-cysteine concentrations [42]. N-Acetyl L-cysteine is a prodrug of L-cysteine and is widely used as a cell-permeable antioxidant [43]. Glutathione tripeptide plays a critical role in antioxidant defense, thiol status maintenance, and xenobiotic detoxification [44]. Previous studies showed that both N-acetyl L-cysteine and glutathione at 5 mM attenuated melatonin-induced melanogenesis in human SK-MEL-1 melanoma cells [45]. Glutathione, glutathione monoethyl ester, and glutathione disulfide whitened the skin to varying degrees [27,46,47]. However, in the current study, L-cysteine, N-acetyl L-cysteine, and glutathione at 0.2–1.0 mM did not reduce melanin content of human melanoma MNT-1 cells, whereas L-cysteinamide reduced the content of melanin (total melanin or eumelanin, but not pheomelanin) at the same concentration range.

L-Cysteine ethyl ester is a hydrophobic prodrug of L-cysteine [48]. N-Acetyl L-cysteinamide is a cell-permeable thiol compound that was developed to enhance cellular glutathione levels [49]. Cysteamine is a metabolite biosynthesized in cells by the degradation of coenzyme A [50]. Of these three compounds, only cysteamine was previously reported to inhibit melanin synthesis in cells [51] and to reduce the melanin content of the melasma lesions in humans [52]. In the current study, L-cysteine ethyl ester and N-acetyl L-cysteinamide did not show antimelanogenic effects at non-toxic concentrations in MNT-1 cells. Cysteamine slightly reduced the melanin content without causing cytotoxicity at 0.5 mM, but it decreased cell viability at 1.0 mM. None of these three compounds were considered to be more useful than L-cysteinamide in developing skin depigmenting agents.

In this study, L-cysteinamide inhibited melanin synthesis in MNT-1 cells more effectively than kojic acid, and as effectively as β-arbutin, without causing cytotoxicity, whereas β-arbutin decreased cell viability, induced morphological changes of cells, and inactivated TYR enzyme in cells. The results support the relative effectiveness and safety of L-cysteinamide as a melanin synthesis inhibitor. L-Cysteinamide could control cellular melanin synthesis without affecting TYR mRNA and protein levels in MNT-1 cells and HEMs.

In understanding the mechanism of action of L-cysteinamide, it will be helpful to review previous studies on L-cysteine in advance. TYR oxidizes pyrocatechol to ortho-quinone, and when L-cysteine is present, a pyrocatechol-cysteine conjugate is generated [26]. When L-tyrosine or L-DOPA is oxidized by TYR, dopaquinone is produced and it is rapidly captured by L-cysteine to form cysteinyldopa, which enters the pheomelanin synthesis pathway. If thiol compounds are not available, dopaquinone spontaneously cyclizes to cyclodopa, which is then oxidized to dopachrome in a reaction coupled with the reduction of dopaquinone to L-DOPA. Dopachrome enters the eumelanin synthesis pathway. By trapping dopaquinone, L-cysteine increases pheomelanin synthesis while decreasing eumelanin synthesis [53].

In previous studies, incubation of human TYR with 10 mM L-cysteine resulted in the inactivation of the enzyme, whereas L-tyrosine or L-DOPA prevented the L-cysteine-induced inactivation by inhibiting the access of L-cysteine to the enzyme active site Cu^2+^ ion [54]. L-Cysteine at 0.1 mM inhibited mouse melanoma TYR hydroxylase activity measured by radioactive water released from L-[3,5-^3^H]-tyrosine substrate (IC_50_, 0.66 mM), as well as the DOPA oxidase activity measured by dopachrome formed [55]. L-Cysteine at 0.15 or 0.30 mM inhibited mushroom TYR activity measured by a spectrophotometry method (IC_50_, 0.15 mM) and a polarography method (IC_50_, 1.44 mM) [56]. In the present study, 200 μM L-cysteine inhibited human TYR-mediated dopachrome formation by 70% (Figure 2b), but no significant change was found in the total melanin content of MNT-1 cells treated with L-cysteine up to 1 mM (Figure 4). Our results are in good agreement with those from another study using human melanoma MM418c5 cells [51]. Therefore, it is suggested that L-cysteine can inactivate the TYR enzyme at a high concentration and inhibit the enzyme activity even at a low concentration, but does not reduce the cellular melanin content at a concentration of up to 1 mM. It is also worth noting that, although L-cysteine and L-cysteinamide share similar chemical properties, their biological activities are not equivalent.

L-Cysteinamide chelated free Cu^2+^ ion in aqueous solution as effectively as kojic acid (Figure 9), which inhibits TYR activity by reversibly binding to Cu^2+^ ion at the enzyme active site [57]. Thus, the Cu^2+^-binding might be a potential mechanism for inhibition of TYR activity by L-cysteinamide. The two Cu^2+^ ions are present at the active site of the TYR enzyme and are each coordinated by three histidine residues [58]. In addition, there are differences in structure and biochemical properties between mushroom and human TYRs [59,60]. Thus, further validation studies are needed to apply this mechanism of action to human TYR.

**Figure 12:**
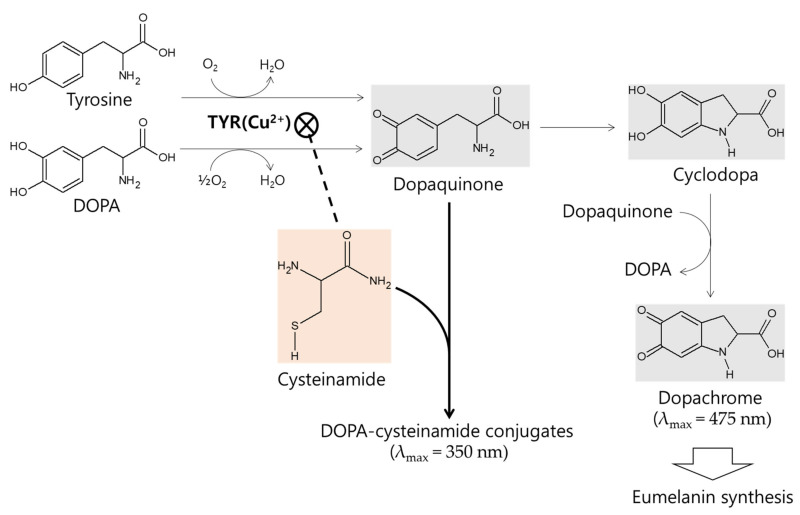
A tentative model for anti-eumelanogenic or anti-melanogenic action of L-cysteinamide. L-DOPA or L-tyrosine is oxidized by TYR to dopaquinone, which is spontaneously converted to cyclodopa and then oxidized to dopachrome. L-Cysteinamide binds the Cu^2+^ ion at the active site of TYR and inhibits its activity. When TYR is partially inhibited by moderate concentrations of L-cysteinamide, dopaquinone synthesis is reduced but still occurs at significant levels. The synthesized dopaquinone is quickly captured by the available L-cysteinamide to form DOPA-cysteinamide conjugates, thus reducing dopachrome formation. If TYR is completely inhibited by high concentrations of L-cysteinamide, no dopaquinone is synthesized, and thus neither DOPA-cysteinamide conjugates nor dopachrome can be produced. Overall, it is proposed that L-cysteinamide may prevent eumelanin synthesis through a dual mechanism by inhibiting TYR activity, thereby reducing dopaquinone synthesis, and by diverting dopaquinone to the formation of DOPA-cysteinamide conjugates, thereby reducing dopachrome formation. At very high concentrations, L-cysteinamide might block total melanin (both pheomelanin and eumelanin) synthesis by almost completely inhibiting TYR activity.

The in vitro kinetic study using mushroom TYR suggests that L-cysteinamide may regulate TYR-mediated dopachrome formation in various ways (Figure 8). L-Cysteinamide at low concentrations (i.e., 100 μM) reduces dopachrome formation by capturing the synthesized dopaquinone to form DOPA-cysteinamide conjugates. L-Cysteinamide at moderate concentrations (i.e., 300 μM) reduces dopachrome formation through a dual mechanism by partially inhibiting TYR activity and by capturing the synthesized dopaquinone. High concentrations (>500 μM) of L-cysteinamide inhibit TYR activity so strongly that neither the DOPA-cysteinamide conjugate nor dopachrome is well formed. A tentative model for anti-eumelanogenic or anti-melanogenic action of L-cysteinamide is proposed in Figure 12.

L-Cysteinamide is considered to regulate the type and total amount of melanin synthesized in cells depending on its concentration. Treatment of MNT-1 cells with 1.0 mM L-cysteinamide resulted in a 39.7% increase in pheomelanin content, a 57.3% decrease in eumelanin content, and a 56.9% decrease in total melanin content (Figure 10). The results suggest that, under this specific condition (we assume that intracellular concentration of L-cysteinamide may be less than 500 μM.), L-cysteinamide may play a dual role as an inhibitor of TYR and a reactant for conjugation reaction with dopaquinone.

To the best of our knowledge, there have been no studies regarding the effects of L-cysteinamide on melanogenesis before the current study. US patent 5,165,427 describes that when reducing agents containing L-cysteinamide or L-cysteine were used for the permanent waving of human hair, the former was about 50% more effective in providing a strong tight curl. This suggests that the chemical properties can vary greatly even by the small structural difference between L-cysteinamide and L-cysteine.

## 5. Conclusions

L-Cysteinamide was found to be one of the most effective anti-eumelanogenic agents among various compounds tested in this study. L-Cysteinamide inhibited TYR-mediated dopachrome formation in vitro and eumelanin synthesis without altering the mRNA and protein expression levels of TYR, TYRP1, and DCT in darkly pigmented human melanoma MNT-1 cells and/or normal HEMs. Thus, L-cysteinamide has great potential for use in controlling skin hyperpigmentation. Further studies are needed to examine its skin depigmenting effects in humans.

## Figures and Tables

**Table 1:** Single letter-codes for twenty different amidated amino acids used in this study.

Single-Letter Codes	Amidated Amino Acids	Single-Letter Codes	Amidated Amino Acids
A-NH_2_	L-Alaninamide hydrochloride	M-NH_2_	L-Methioninamide hydrochloride
C-NH_2_	L-Cysteinamide hydrochloride	N-NH_2_	L-Asparaginamide hydrochloride
D-NH_2_	L-Aspartic acid α-amide hydrochloride	P-NH_2_	L-Prolinamide hydrochloride
E-NH_2_	L-Glutamic acid α-amide	Q-NH_2_	L-Glutaminamide hydrochloride
F-NH_2_	L-Phenylalaninamide hydrochloride	R-NH_2_	L-Argininamide dihydrochloride
G-NH_2_	L-Glycinamide hydrochloride	S-NH_2_	L-Serinamide hydrochloride
H-NH_2_	L-Histidinamide dihydrochloride	T-NH_2_	L-Threoninamide hydrochloride
I-NH_2_	L-Isoleucinamide hydrochloride	V-NH_2_	L-Valinamide hydrochloride
K-NH_2_	L-Lysinamide dihydrochloride	W-NH_2_	L-Tryptophanamide hydrochloride
L-NH_2_	L-Leucinamide hydrochloride	Y-NH_2_	L-Tyrosinamide hydrochloride

**Table 2:** Sequences of primers used for quantitative reverse transcriptase-polymerase chain reaction (qRT-PCR).

Genes	GenBank Accession Number	Forward (F) and Reverse (R) Primer Sequences	References
*TYR*	NM_000372.5	F: 5′-GCCAACGATCCTATCTTCCTTC-3′ R: 5′-GTGCATTGGCTTCTGGATAAAC-3′	[36]
*TYRP1*	NM_000550.3	F: 5′-GCTTTTCTCACATGGCACAG-3′ R: 5′-GGCTCTTGCAACATTTCCTG-3′	[37]
*DCT*	NM_001129889.3	F: 5′-TGCATTTGTTACCTGGCACC-3′ R: 5′-ATCACACTCGTTCCTCCCAG-3′	[38]
*GAPDH*	NM_001357943.2	F: 5′-GACCACTTTGTCAAGCTCATTTC-3′ R: 5′-CTCTCTTCCTCTTGTGCTCTTG-3′	[36]

## Data Availability

The data is contained within the article.

## Abbreviations

DCT	dopachrome tautomerase
DMSO	dimethyl sulfoxide
DOPA	dihydroxyphenylalanin
GAPDH	glyceraldehyde 3-phosphate dehydrogenase
HEM	human epidermal melanocyte
PCV	pyrocatechol violet
qRT-PCR	quantitative reverse transcription-polymerase chain reaction
TYR	tyrosinase
TYRP1	tyrosinase-related protein 1
